# Anadromy, potamodromy and residency in brown trout Salmo trutta: the role of genes and the environment

**DOI:** 10.1111/jfb.14005

**Published:** 2019-06-13

**Authors:** Andrew Ferguson, Thomas E. Reed, Tom F. Cross, Philip McGinnity, Paulo A. Prodöhl

**Affiliations:** ^1^ School of Biological Sciences Queen's University Belfast Belfast UK; ^2^ School of Biological, Earth and Environmental Sciences University College Cork Cork Ireland

**Keywords:** allacustrine, fluvial–adfluvial, lacustrine–adfluvial, physiological condition, threshold trait

## Abstract

Brown trout Salmo trutta is endemic to Europe, western Asia and north‐western Africa; it is a prominent member of freshwater and coastal marine fish faunas. The species shows two resident (river‐resident, lake‐resident) and three main facultative migratory life histories (downstream–upstream within a river system, fluvial–adfluvial potamodromous; to and from a lake, lacustrine–adfluvial (inlet) or allacustrine (outlet) potamodromous; to and from the sea, anadromous). River‐residency *v*. migration is a balance between enhanced feeding and thus growth advantages of migration to a particular habitat *v*. the costs of potentially greater mortality and energy expenditure. Fluvial–adfluvial migration usually has less feeding improvement, but less mortality risk, than lacustrine–adfluvial or allacustrine and anadromous, but the latter vary among catchments as to which is favoured. Indirect evidence suggests that around 50% of the variability in S. trutta migration *v*. residency, among individuals within a population, is due to genetic variance. This dichotomous decision can best be explained by the threshold‐trait model of quantitative genetics. Thus, an individual's physiological condition (*e.g*., energy status) as regulated by environmental factors, genes and non‐genetic parental effects, acts as the cue. The magnitude of this cue relative to a genetically predetermined individual threshold, governs whether it will migrate or sexually mature as a river‐resident. This decision threshold occurs early in life and, if the choice is to migrate, a second threshold probably follows determining the age and timing of migration. Migration destination (mainstem river, lake, or sea) also appears to be genetically programmed. Decisions to migrate and ultimate destination result in a number of subsequent consequential changes such as parr–smolt transformation, sexual maturity and return migration. Strong associations with one or a few genes have been found for most aspects of the migratory syndrome and indirect evidence supports genetic involvement in all parts. Thus, migratory and resident life histories potentially evolve as a result of natural and anthropogenic environmental changes, which alter relative survival and reproduction. Knowledge of genetic determinants of the various components of migration in S. trutta lags substantially behind that of Oncorhynchus mykiss and other salmonines. Identification of genetic markers linked to migration components and especially to the migration–residency decision, is a prerequisite for facilitating detailed empirical studies. In order to predict effectively, through modelling, the effects of environmental changes, quantification of the relative fitness of different migratory traits and of their heritabilities, across a range of environmental conditions, is also urgently required in the face of the increasing pace of such changes.

## 1 INTRODUCTION

Migration occurs in all major animal taxa and results from spatial, seasonal and ontogenetic separation of optimal habitats for feeding and breeding (Northcote, 1984). However, the spatial patterns and behaviours involved vary enormously among species, populations and individuals (Dingle & Drake, 2007). Better understanding of migration requires studies of convergent processes across a wide range of taxa (Dingle, 2014; Sahashi & Morita, 2013). In broad terms, the study of migratory syndromes, the integrated suites of traits, behaviours and physiological processes involved directly or indirectly in migration (Dingle, 2006; van Noordwijk *et al*., 2006), can be approached from both proximate and ultimate perspectives (Tinbergen, 1963). Proximate questions concern how migratory tendencies, behaviours or associated traits are expressed in individuals in response to environmental cues or constraints during ontogeny. Ultimate questions focus instead on the evolutionary functions and phylogenetic history of migration. The proximate mechanisms themselves, however, have evolved in response to past environmental pressures and can evolve further as selective regimes change. In recent years there has been an increasing realisation that genetic mechanisms play a major role in the control of migratory behaviour in a wide range of animals and that a study of this genetic architecture enhances our understanding of the mechanisms involved (Liedvogel *et al*., 2011). It is also essential to understand how natural selection operates at various levels in the complex chain linking genes to phenotypes to Darwinian fitness in variable environments. These insights can then feed into a more evolutionarily‐enlightened approach to the conservation and management of migratory species, which face multiple anthropogenic threats worldwide.

A crucial, but surprisingly understudied, aspect of migration biology concerns the migratory decision (Dingle & Drake, 2007; Pulido, 2011). While some species have obligate migratory or non‐migratory life histories, others exhibit intraspecific variation in migratory tendencies, with populations in some parts of the range being fully migratory, others being fully resident and yet others exhibiting a facultative mix of migratory and resident individuals (‘partial migration’ of some authors; Chapman *et al*., 2011). Fishes provide many interesting examples here, both in terms of population and individual‐level variation in migratory tendencies, but also in the habitats and environments to which fish migrate (Chapman *et al*., 2012). Salmonids are particularly interesting in this regard as they can exhibit large or short distance migrations or fully resident life histories (Dodson *et al*., 2013). Migrations can be between fresh water and salt water or confined to lakes and rivers. Like any complex phenotype, variation in migratory strategies reflects interaction between genetic and environmental influences (Pulido, 2011), with the relative importance of genes and environment probably varying across different phenotypic components of an overall life history strategy (van Noordwijk *et al*., 2006).

Although from three to 50 species of trout of the genus *Salmo* L. 1758 are currently recognised by some authorities (Froese & Pauly, 2019; Kottelat & Freyhof, 2007; Whiteley *et al*., 2019), for the purposes of this review it is treated, *sensu lato*, as brown trout *Salmo trutta* L., since information from Adriatic softmouth trout *Salmo obtusirostris* (Heckel 1851) and Ohrid belvica *Salmo ohridanus* Steindachner 1892, which are regarded by Whiteley *et al*. (2019) as valid species, is not included here. *Salmo trutta* are native to Europe and western Asia, together with a small number of populations in north‐western Africa, although many natural populations are now extinct (Ferguson *et al*., 2019; Lobón‐Cerviá *et al*., 2019; Markevich & Esin, 2019; Rasmussen *et al*., 2019; Schöffmann *et al*., 2019). *Salmo trutta* are arguably one of the most diverse salmonids in terms of their morphologies, life histories and migratory tactics (Klemetsen 2013). This review complements that of Ferguson *et al*. (2017), which it updates extensively and extends to include potamodromy and a more detailed consideration of genetic aspects, including evolutionary responses to changing environmental conditions. While information relates, where possible, to the entire native range of *S. trutta*, most studies have been carried out in north‐western Europe. Where information is lacking for *S. trutta*, or where strong supporting evidence is available, comparative information is used from studies on other salmonines, in particular rainbow–steelhead trout *Oncorhynchus mykiss* (Walbaum 1792). Such information also serves to highlight gaps in the knowledge of genetics of *S. trutta* migration and emphasise areas where research could be undertaken profitably (see §8). Since salmonines within the sub‐family comprising *Salmo*, *Oncorhynchus* Suckley 1861, *Salvelinus* Richardson 1836 and *Parahucho* Vladykov 1963 all share the potential for migratory behaviour (Alexandrou *et al*., 2013) there is much opportunity for comparative studies. Where the term salmonines is used below it is the sub‐family overall that is referred to and the characteristic has been shown to, or is likely to, occur in several species.

### 1.1 Terminology

**Figure 1:**
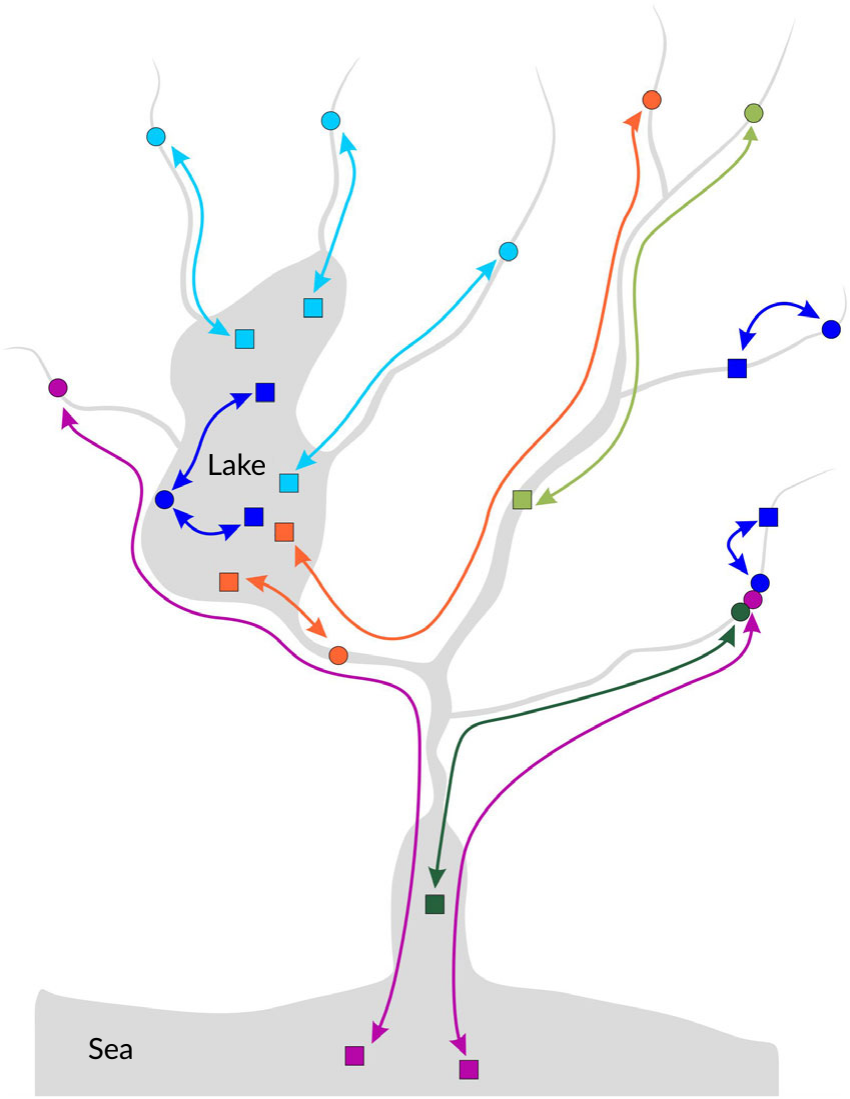
Potential life‐history diversity of Salmo trutta in a typical catchment with a lake. 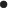, Spawning locations; 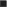, adult feeding sites. (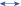) Lake‐ or river‐resident, (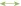) Fluvial–adfluvial, (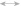) Lacustrine–adfluvial, (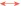) Allacustrine, (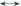) Semi‐anadromous, and (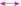) Anadromous

In *S. trutta* there are two resident and three main migratory life histories (Figure 1), although considerable subdivision of these categories is possible when precise destination and life‐history details, including age and timing of various events and repeat spawning, are taken into account (Huusko *et al*., 2018)*. Salmo trutta* can be resident within rivers, often in a 1st or 2nd order tributary, for their entire life cycle; *i.e*., river‐resident. Included within this term are individuals that make early localised dispersal movements, as described for example by Vøllestad *et al*. (2012)*. Salmo trutta* can also be resident within lakes with their entire life cycle being spent there; *i.e*., lake‐resident. This life history is probably more common than hitherto recognised. However, there may be both horizontal and vertical movements within lakes between spawning and feeding grounds and on a diurnal basis (Jonsson & Jonsson, 2018). Lake‐spawned *S. trutta* appear to remain resident and do not migrate to the river or sea, as, for example, occurs with some sockeye salmon *Oncorhynchus nerka* (Walbaum 1792); although this aspect has not been specifically investigated. Some authors use the term freshwater‐resident in the sense of river‐resident only, while others use it in the sense of inhabiting to include all freshwater forms including migratory ones. Because of this ambiguity, the term should not be used but, instead, precise life history should be specified (Ferguson *et al*., 2017).

In this review migration refers to directed movements between two distinct habitats occurring with regular periodicity on a temporally predictable basis (Brönmark *et al*., 2014; Northcote, 1978). Out‐migration typically takes place for feeding, or to find temporary refuge. Thus, extreme temperature and water flow in the natal river can result in *S. trutta* moving downstream to find refuge, presumably as a direct result of stress. Out‐migration is followed by a return migration to the place of natal origin for subsequent spawning, or to the natal or non‐natal area for refuge from harsh conditions (Klemetsen *et al*., 2003; Jensen *et al*., 2015). The terminology used here for potamodromous (freshwater) migrations follows that of Varley and Gresswell (1988) as elaborated by Northcote (1997)*. Salmo trutta* resulting from spawning in rivers can undergo three main types of migration to feeding areas (Figure 1) and subsequent adult return, involving: (a) a larger tributary or typically the main stem of the river, fluvial–adfluvial migration; (b) a lake, lacustrine–adfluvial migration if an inlet river is involved, or allacustrine migration where outlet river spawning occurs; and (c) the sea, anadromy. In the latter case movement may only be as far as the estuary, with some authors referring to this as semi‐anadromy or partial‐anadromy, but, confusingly, these terms are also used by other authors in the sense of facultative migration. In Denmark and Norway migration can be terminated in a fjord rather than continue to the open sea (del Villar‐Guerra *et al*., 2014; Thorbjørnsen *et al*., 2018). Some authors (Quinn, 2018) use a more abbreviated terminology for potamodromy simply referring to adfluvial for migrations between natal rivers and lakes and fluvial both for migrations within rivers and river‐residents.

In the literature there are many references to migratory *S. trutta* in the sense of anadromy only. However, potamodromous migrations are widespread and in many parts of the range such migrations are more numerous or are the only migrations present. A fundamental thesis of this review is that fluvial–adfluvial, lacustrine–adfluvial, allacustrine and anadromous migrations are fundamentally the same as to their determinants and thus information on one form of migration is relevant to the others. It is important, however, in this context to separate the decision to migrate from the decision as to the destination of migration. The exclusive focus on anadromy in many studies is probably due to the commercial and recreational importance of anadromous *O. mykiss* in western North America and anadromous *S. trutta* in north‐western Europe, where most studies have been undertaken, rather than any major difference in their migration. Comparatively few studies have been carried out into the determinants of lacustrine–adfluvial and allacustrine migrations in salmonines and even less on fluvial–adfluvial migration. This bias is inevitably reflected in the relative coverage here. Here the term migrant is used where all types of migration are being referred to but otherwise qualified. Due to the considerable similarity in determinants and processes outlined below and in Table 1, the term smolt is not restricted to destinations involving hypo‐osmoregulation and is used here for all downstream migrating juveniles irrespective of their ultimate destination, as has also been applied by other authors (Huusko *et al*., 2018; Jones *et al*., 2015) and indeed has been widely used for migratory *S. trutta* in the Baltic Sea.

**Table 1:** Similarities between lacustrine–adfluvial and anadromous life histories for various characteristics in Salmo trutta and other salmonines, together with characteristics* observed in fluvial–adfluvial migrants also (which have been less extensively studied than the other two life histories)

Characteristic	Reference (s)
Increased growth*	Ayer *et al*., 2017; Brönmark *et al*., 2014
Increased mortality*	Healy *et al*., 2017; Schwinn *et al*., 2018
Sex ratio biased towards females*	Ayer *et al*., 2017; García‐Vega *et al*., 2018; Huusko *et al*., 2018
Downstream movements occurs at the same time in spring*	Ayer *et al*., 2017; Holecek & Scarnecchia, 2013; Pirhonen *et al*., 1998
Changes in body shape; longer but thinner*	Ayer *et al*., 2017
Silvery body colour	Authors’ observations
Increase in NKA activity*	Boel *et al*., 2014; Inatani *et al*., 2018
Retention of genetic differences associated with osmoregulation	Arostegui *et al*., 2019
Transaldolase 1 and endozopine are expressed at lower levels some 3 months prior to migration	Amstutz *et al*., 2006; Giger *et al*., 2006, 2008
Outlier SNPs mapped to genes *znf665*‐like, *grm4*‐like, *pcdh8*‐like, & *st3gal1*‐like	Lemopoulus *et al*., 2018
*Oncorhynchus mykiss* migration is associated with MAR region on chromosome *Omy5*	Arostegui *et al*., 2019; Kelson *et al*., 2019; Leitwein *et al*., 2017; Pearse & Campbell 2018

The differences in characteristics are relative to the river‐resident tactic.

MAR: migration‐associated region; NKA: Na^+^K^+^‐ATPase; SNP: single nucleotide polymorphism.

### 1.2 Life‐history occurrence and patterns

In the formerly glaciated region of north‐western Europe, as a result of marine barriers after the ice retreated, most current freshwater *S. trutta* populations are derived from anadromous ancestors (Ferguson, 2006). Clearly this anadromous life‐history trait is not fixed in *S. trutta* but can change over time given that many potamodromous and resident populations are now present in this region. Populations from different parts of the range of *S. trutta*, even geographically adjacent ones, often differ markedly in life‐history characteristics as a result of differences in factors such as phylogeographic origin (McKeown *et al*., 2010), current environmental conditions including both abiotic and biotic factors and ecological opportunity such as nutrient richness of different habitats (Jonsson & Jonsson, 2018). River‐resident *S. trutta* can occur facultatively within rivers with open access to both out and return‐migration and obligately where there are barriers to return migration as a result of waterfalls and artificial dams. In the southern part of the range, high temperature and river‐flow regimes can likewise form a barrier to downstream migration with populations confined to headwaters (García‐Marín *et al*., 2018). Spawning of lake‐resident *S. trutta* occurs on shoreline gravels where there is sufficient wave action or diffuse water flow from the surrounding land to provide oxygenation. This type of spawning is typical of many small upland lakes (Prodöhl *et al*., [Link]). Spawning has been shown to occur in gravel areas in large lowland Irish lakes such as Lough Melvin (54° 24′ N, 08° 07′ W; Ferguson & Taggart, 1991) and Lough Mask (53° 36′ N, 09° 22′ W; P. Gargan, Inland Fisheries Ireland, pers. comm.). Spawning can also occur deep within lakes where there is upwelling from bottom fissures. The latter is typical of spawning in many volcanic‐region lakes in Iceland where water flow is underground (Ferguson *et al*., 2019) and in lakes in limestone areas elsewhere. In Lake Garda (Italy; 45° 34′ N, 10° 38′ E), native *S. trutta* spawning has been shown to occur at 200–300 m depth and in Lake Posta Fibreno (Italy; 41° 41′ N, 13° 41′ E) spawning occurs in underground spring‐fed karstic pools (Meraner & Gandolfi, 2018).

Fluvial–adfluvial, lacustrine–adfluvial and allacustrine migratory *S. trutta* occur throughout the native range where suitable conditions exist. Genetic assignment studies have indicated that *S. trutta* feeding in the mainstems of some Irish rivers are entirely composed of recruits from tributaries; *i.e*., they are fluvial–adfluvial migrants (Ferguson *et al*., 2019). In regions such as Ireland and Scotland, UK, with thousands of freshwater lakes, a lacustrine–adfluvial life history is numerically the most common one, based on the relative abundance of such populations (Ferguson *et al*., 2019). Allacustrine populations are also widespread and are typically reproductively isolated and genetically distinct from the lacustrine–adfluvial populations of the same lake (Ferguson, 2004). Since outlet rivers have more often been modified by damming, drainage *etc*., than inlet ones, allacustrine populations have been adversely affected. For example, in Finland allacustrine populations are mostly extinct or extremely endangered (Syrjänen *et al*., 2018). Anadromous *S. trutta* are found in western Europe from the Mondego River in central Portugal (Caballero Javierre *et al*., 2018) northwards to Scandinavia and the Cheshkaya Gulf in north‐western Russia, including Iceland and the Baltic Sea (Klemetsen *et al*., 2003), although natural stocks in Finnish and Polish rivers have largely been lost (Dębowski, 2018; Kallio‐Nyberg *et al*., 2010; Soininen *et al*., 2018). Anadromous *S. trutta* are found in the Black and Caspian Sea drainages (Makhrov *et al*., 2018) and formerly also in the Aral Sea prior to desiccation and salinity increase (Markevich & Esin, 2019). Anadromous *S. trutta* are currently absent from Mediterranean rivers, most likely because of the high temperature of the sea, although the widespread distribution of *S. trutta* in many unconnected catchments indicates that anadromy occurred during glacial periods when the sea temperature was lower (Gibertoni *et al*., 2014).

Major physiological differences between potamodromy and anadromy are the changes required for osmoregulation and ionic regulation. Thus, in fresh water the body fluids of *S. trutta* and other teleosts are hyperosmotic and hypotonic to the surrounding water and are faced with the gain of water by osmosis and the loss of ions by diffusion, with the reverse being the case in full‐strength seawater. Anadromy thus requires a change in regulation when moving between fresh and seawater to maintain osmotic and ionic homeostasis. This is achieved through osmosensing, which is the physiological process of perceiving a change in environmental salinity and with which many genes have been found to be associated (Kültz, 2013). Teleosts maintain their internal salt concentration at around one‐quarter to one‐third of full‐strength seawater; *i.e*., 0.9–1.1% (Edwards & Marshall, 2013). Thus, the distinction is between hyper‐osmoregulation at < *c*. 1% salinity and hypo‐osmoregulation at > *c*. 1% salinity, irrespective of geographical quirks of naming water bodies. In the Baltic Sea, salinity in surface waters, except close to the Kattegat, is generally < 1%, (HELCOM, 2018). The salinity of the Caspian Sea ranges from 0.1% in the north to 1.35% in the south (Caspinfo, 2018). Thus, in much of the Baltic and Caspian Seas, hyper‐osmoregulation occurs as in fresh water. This is also shown by the fact that *S. trutta* spawning can occur in the Baltic Sea (Landergren & Vallin, 1998). Fry and 0+ year‐old parr can migrate to the Baltic Sea (Landergren, 2001) and such juveniles can further migrate into non‐natal streams before later becoming smolts and descending to the sea again (Taal *et al*., 2018).

Migratory behaviour can change within the lifetime of individuals, which further indicates the lack of a clear separation among migratory life histories. Formerly anadromous *S. trutta* can subsequently adopt a freshwater life history (Klemetsen *et al*., 2003), a phenomenon also known in *O. mykiss* (Null *et al*., 2013) and dolly varden charr *Salvelinus malma* (Walbaum 1792), where older individuals cease to migrate (Bond *et al*., 2015). It is increasingly recognised that anadromous *S. trutta* can spend a lesser part of their life at sea with the rest spent in lakes or rivers. In a Norwegian tracking study involving previously spawned migrants (kelts), variation in marine residence ranged from 7 to 183 days, this residence being positively correlated with size and original smolt age and negatively with date of sea entry (Eldøy *et al*., 2015). In Loch Lomond (Scotland; 56° 05′ N, 04° 36′ W), a 71 km^2^ freshwater lake, carbon stable‐isotope analysis showed that individual *S. trutta* appear to move repeatedly between the lake and estuarine–marine environments (Etheridge *et al*., 2008). In this case there is only a short river (*c*. 10 km) separating the loch and the Clyde Estuary and it may be that migration does not go beyond the estuary. In the Näätämöjoki river system in northern Scandinavia (60° 42′ N, 29° 05′ E), Ruokonen *et al*. (2018) found mainly two distinct groups based on carbon stable‐isotope analysis, but with some individuals showing intermediate values suggesting repeated movements between fresh water and the sea.

## 2 WHY MIGRATE AND WHERE?

*Salmo trutta* populations in many rivers show facultative migration, with part of the population migrating while other individuals remain resident within their natal river. Migration potentially offers many benefits to individuals while at the same time these are countered by ensuing costs (Brönmark *et al*., 2014; Gross, 1987; Quinn & Myers, 2004), resulting in the outcome being finely balanced between these conflicting aspects (Ferguson *et al*., 2017). Advantages and disadvantages are likely to vary among populations and temporally as a result of environmental and biotic changes; *e.g*., population density. The increasing food availability hypothesis (Gross *et al*., 1988) explains why salmonines migrate from natal areas, with a balance of relative risks and rewards determining where they migrate to. Migration downstream within a river system, to a lake, or to the sea probably increases feeding opportunities. Mechanisms driving these migrations are probably the same as long as productivity between natal river and feeding habitats is significantly different (Ayer *et al*., 2017). Better feeding, both in terms of quantity and quality, results in faster growth, potentially larger size at maturity, higher fecundity, greater energy stores at reproduction and thus more offspring are produced (Acolas *et al*., 2008; Fleming & Reynolds, 2004; Jonsson & Jonsson, 2006a). Goodwin *et al*. (2016) found that the parental contribution of males and, especially, females to the juvenile production in a river was much higher for anadromous than river‐resident *S. trutta*.

Based on size at maturity, lake and sea feeding is superior to remaining within the river, albeit the relative importance of lake and sea feeding varies among catchments. If lacustrine *S. trutta* become piscivorous (Campbell, 1979; Wollebaek *et al*., 2018), they can reach a larger size than anadromous conspecifics. Thus, in both Britain and Ireland, the largest rod‐caught piscivorous lacustrine *S. trutta* to date have had a greater mass than the largest anadromous *S. trutta* (Ferguson *et al*., 2017), although the abundance of prey fish is such that only a small proportion of individuals can adopt piscivory (Campbell, 1979; Hughes *et al*., 2016), compared with the greater abundance of prey fish at sea. However, in Finland, where the prey consists of abundant European whitefish *Coregonus lavaretus* (L. 1758), vendace *Coregonus albula* (L. 1758) and European smelt *Osmerus eperlanus* (L. 1758), almost all lacustrine *S. trutta* are piscivorous (Huusko *et al*., 2018). The largest *S. trutta* known were the so‐called salmon of the Caspian Sea with the largest recorded being 57 kg (Markevich & Esin, 2019), although their size has been decreasing in recent decades (Niksirat & Abdoli, 2009). Large *S. trutta* are also known from the Baltic Sea (Rasmussen & Pedersen, 2018). Possibly, the brackish nature of these seas results in less energy expenditure for osmoregulation than in either fresh water or full‐strength seawater (see §1.1).

On the adverse side, migration increases energy expenditure, physiological stress, risk of predation, parasites and diseases, both during migration and in the subsequent habitat. Migration downstream within a river system, to a lake, or to the sea increases risk in that order. The number of *S. trutta* predators appears to be higher at sea than in fresh water (Jonsson & Jonsson, 2004) and predation is a major mortality factor shortly after smolts reach the sea (Dieperink *et al*., 2002; Healy *et al*., 2017). Predation in lakes is generally higher than that in rivers (Schwinn *et al*., 2018) and especially at river–lake confluences (Kennedy *et al*., 2018).

Lacustrine–adfluvial migration is probably similar or better in benefits to anadromy in some cases but lowers the relative costs due to lowered energy expenditure and decreased risk of predation. Anadromous *S. trutta* occur especially in shorter river systems of low alkalinity with good spawning and nursery areas easily accessible from the sea and especially where river or lake productivity is low (CSTP, 2016). In higher productivity lakes, lacustrine–adfluvial migration can occur exclusively even where there is no barrier to anadromy suggesting that, in that situation, it is superior in terms of cost–benefit considerations. In other cases, both lacustrine–adfluvial and anadromous *S. trutta* are present in the same catchment, with the lacustrine–adfluvial form often predominating (Poole *et al*., 2006). In *O. mykiss*, anadromy is also less common in river systems with large lakes (Kendall *et al*., 2015). In some situations, *S. trutta* migration may occur to downstream brackish lakes or estuaries. Thus, in the Burrishoole system (western Ireland) many smolts migrate to the brackish Lough Furnace (53° 55′ N, 09° 35′ W) and appear to remain there, or in the estuary, before returning to fresh water (Poole *et al*., 2006). It has been suggested that estuaries provide better feeding than rivers but with reduced likelihood of predation and reduced salinity compared with the open sea (Thorpe, 1994), although fluctuating salinity may actually produce greater physiological stress than the higher, but more stable, salinity of seawater (Jensen & Rikardsen, 2012).

### 2.1 Sex and facultative migration

Many studies have shown that in *S. trutta* populations there is generally a sex bias, with typically females predominating among fluvial–adfluvial, lacustrine–adfluvial and anadromous migrants and males among residents (Ayer *et al*., 2017; Ferguson *et al*., 2017; García‐Vega *et al*., 2018; Huusko *et al*., 2018). A sex bias is to be expected from the balance of benefits of migration and residency (Hendry *et al*., 2004). Thus, female reproductive success is generally limited by gamete production with a larger body size giving greater fecundity and egg size (Fleming, 1996; Quinn, 2018). Larger females can attract mates, acquire and defend better spawning sites in a wider range of substrate sizes and excavate deeper nests (Fleming & Reynolds, 2004).

Compared with females, male reproductive success is typically limited by access to mates (Fleming, 1998) rather than gamete production, since even small males can produce millions of sperm (Munkittrick & Moccia, 1987). While a larger size can be of benefit to males in attracting and defending mates, obtaining a large body size is less critical for male reproduction because instead of aggressive defence of females, a tactic typically displayed by larger anadromous males (Esteve, 2005), they can adopt a sneaking tactic allowing successful egg fertilisation at a small size (Gross, 1985). Thus, males more often mature as residents since they are less dependent on large body size for reproductive success and, consequently, mature across a much greater range of ages and sizes (Jonsson & Jonsson, 1993). Early maturity in males also results in reduced pre‐reproductive mortality (Gross & Repka, 1988). In Atlantic salmon *Salmo salar* L. 1758, male parr may mature while still in fresh water and then subsequently undergo an anadromous life history (Mitans, 1973). In *S. trutta*, maturation appears to exclude subsequent anadromy (Dębowski & Dobosz, 2016). However, L'Abée‐Lund *et al*. (1990) note that the same individuals can mature both as parr and later as sea‐run adults, although limited evidence is provided. Clearly this aspect requires detailed investigation in other populations.

Differential sexual manifestation of migration and residency reflects genetic control since the environment experienced by the sexes is assumed to be the same. Co‐regulation of both sex‐specific and autosomal genes can be involved, acting through hormonal and epigenetic regulation (Sutherland *et al*., 2018). Hale *et al*. (2018) measured sex‐bias in gene expression in the brain transcriptome of *O. mykiss* in two F_1_ lines derived from migratory and resident fish, which were reared in a common‐garden environment to reveal heritable differences. The parents came from Sashin Creek (Alaska; 56° 21′ N, 134° 43′ W), the residents being from an above‐waterfalls population that had been artificially established from the anadromous stock below the waterfalls some 70 years previously (Thrower *et al*., 2004). Overall 1716 genes (4.6% of total examined) showed evidence of sex‐biased gene expression involving at least one time point from the fry stage through to when they either migrated to the ocean or remained resident and became sexually mature. The majority (96.7%) of sex‐biased genes were differentially expressed during the second year of development, indicating that patterns of sex‐bias in expression are linked to key developmental events, such as migration and sexual maturation. This is not surprising as the brain is involved in hormonal regulation of both of these processes (Hale *et al*., 2018). Most of the sex‐biased expression was in the migratory line, which is likely to have included both migrant and resident individuals, with life‐history choice being different for the sexes. The lack of sex bias in the resident line suggests similar developmental pathways to residency and sexual maturation in both males and females.

Differential sexual expression of migration with a common genetic basis results in sexual conflict; *i.e*., alleles conferring higher reproductive success in one sex can decrease the fitness of the other sex (Chapman *et al*., 2003). In *O. mykiss*, a 56 Mb double‐inversion and hence recombination protected, gene complex on *O. mykiss* chromosome 5 (*Omy5*) facilitates sex‐specific migration through asymmetric sex‐dependent dominance, thus reducing sexual conflict (Pearse *et al*., 2018). Karyotypes at the *Omy5* double inversion were classified as ancestral (A) and rearranged (R) relative to other salmonines, with both karyotype and sex influencing the tendency to migrate. Females of AA and AR genotype were equally likely to migrate (complete dominance) with the tendency being twice that of RR females. Heterozygous (AR) males were more similar to RR males in their tendency to be resident (partial dominance). This supergene complex contains many genes known to be associated with key life‐history traits including photoperiod perception, circadian rhythms, age of maturation, energy storage and sex determination (Pearse *et al*., 2018). To date, there are no published studies looking for the equivalent of the *Omy5* region in *S. trutta*, although a few studies have looked at genes associated with some of these traits (Lemopoulos *et al*., 2018 – see §3.1 and Table 1).

## 3 GENETIC AND ENVIRONMENTAL DETERMINANTS OF FACULTATIVE MIGRATION

### 3.1 Reproductive isolation and heritability

The key behavioural step for a young *S. trutta* in its natal river is whether to remain in the river and become sexually mature or migrate to a higher‐order tributary, a lake, or the sea. While offspring of migratory and river‐resident *S. trutta* can show different life histories from their parents, there is often a strong tendency to track the parental life history (Dębowski & Dobosz, 2016; Jonsson, 1982; Skrochowska 1969). Berejikian *et al*. (2014) showed that female offspring produced by anadromous *O. mykiss* mothers rarely expressed residency (2%), while the percentage of maturing male parr produced was much higher (41%) across a diversity of freshwater habitats. Also, both male and female parr that were produced by resident mothers were significantly more likely to show residency than the offspring of anadromous mothers. Female body size has a significant effect on egg size, a heritable trait (Carlson & Seamons, 2008), which affects survival and growth of juveniles, especially in the early stages of life (Thorn & Morbey, 2018). Associations between maternal and offspring life‐histories could therefore reflect a mix of direct genetic effects (*e.g*., where offspring inherit migratory alleles from their mother), indirect genetic effects (*e.g*., where genes carried by the mother affect the size of her eggs, which in turn influences offspring life history) and maternal environmental effects (where environmental effects on the mother's phenotype influence offspring life history).

Most studies with neutral genetic markers have failed to find genetic differentiation between strictly sympatric (syntopic) migratory and river‐resident *S. trutta* (Ferguson *et al*., 2017). However, this does not imply that the difference between these life histories does not have a genetic basis, only that it is not population based. Thus, most genetically‐based characteristics are inherited through family lineages. There are many cases of neutral genetic markers showing genetically distinct resident *S. trutta* above complete or partial barriers that are impassable or have restricted passage to upstream migration, compared with migratory ones below, as would be expected from allopatric populations where gene flow is limited or absent (Ferguson, 1989). In addition, there are also situations of upstream resident and downstream facultative migratory *S. trutta* populations within the same river but without any physical barrier to upstream movement (Lemopoulos *et al*., 2017), with the two types representing separate colonising lineages in some cases (Hamilton *et al*., 1989; McKeown *et al*., 2010; Turan *et al*., 2009). In some situations of sympatric differentiation below barriers, the resident *S. trutta* or *O. mykiss* appear to have arisen from displacement of such fish from above a barrier where strong selection for residency is expected (Ferguson *et al*., 2017; Pearse *et al*., 2009). Where migratory and river‐resident salmonines occur in syntopy, behavioural or temporal differences in spawning could result in reproductive isolation.

Overall the evidence suggests that there is a strong genetic element involved in facultative migration in salmonines (Ferguson *et al*., 2017; Kendall *et al*., 2015). Thrower *et al*. (2004) bred pure and reciprocally‐crossed lines of anadromous and resident *O mykiss* from Sashin Creek. After 2 years in a communal hatchery environment, they found that narrow sense heritability (h^2^: additive genetic variance only as a fraction of phenotypic variance) estimates for freshwater maturation and anadromy were 0.44–0.51 and 0.45–0.56 respectively. Hecht *et al*. (2015) found a modal h^2^ estimate of 0.61 (0.39–0.77) for life history in the same hatchery lines but using a larger pedigree. They also found significant genetic correlations of life history with growth rate, size at age, condition factor and morphological traits, which themselves showed moderate heritabilities. Doctor *et al*. (2014) reciprocally transplanted two populations of anadromous *O. mykiss* from cooler and warmer conditions. Although there were strong genotype‐temperature interactions broad‐sense estimates of heritability (H^2^: all genetic variance) for the anadromy decision were very similar at 0.69 and 0.77. Heritability (h^2^) estimates for anadromy in a natural population of brook charr *Salvelinus fontinalis* (Mitchill 1814) were 0.52–0.56 (Thériault *et al*., 2007). While no heritability estimates have yet been published for *S. trutta* migration, the similarity of *O. mykiss* and *S. fontinalis* estimates under very different environments, in spite of the few populations examined, may suggest heritability of a similar magnitude. In addition, 20 heritability (h^2^) estimates for migratory traits in birds gave a mean value of 0.37 (± SD = 0.23; Pulido, 2007). Thus, in common with other threshold traits (Dodson *et al*., 2013; Roff, 1996), it is likely that around half of the phenotypic variability in *S. trutta* migration *v*. residency among individuals within a population is due to additive genetic variance, with the remainder attributed to non‐additive genetic variance, non‐genetic parental effects and environmental influences. However, it is very important to acknowledge that heritability estimates are specific to the population and particular environmental conditions examined. Explicit estimates are required for a range of *S. trutta* populations under different conditions before credence is given to any estimate. The same also applies to genetic correlations among traits and it may be the case that patterns of phenotypic integration (when multiple functionally‐related traits are correlated with each other, in part due to pleiotropic effects of genes) may be quite different in *S. trutta* compared with *O. mykiss*, with some aspects being less restricted to evolve independently than others.

Studies of the genetic basis of anadromy have identified several gene markers and chromosome regions associated with migration and residency in *O. mykiss*. The large inversion complex on *Omy5*, noted in §2.1 and which, in a population context, has been referred to as the migration‐associated region (MAR), is of particular interest. Based on screening of two linked single nucleotide polymorphisms (SNP), MAR shows alternative genotypes that are strongly associated with either migration or river‐residency (Hecht *et al*., 2013; Leitwein *et al*., 2017; Pearse *et al*., 2014). Kelson *et al*. (2019) found that a combination of MAR genotype and genetic sex predicted 45% of the life‐history variation in *O. mykiss*, but that resident genotypes could give rise to migrants. MAR genes have been associated with important traits including smoltification, growth rate, developmental rate, survival in seawater and out‐migration of juveniles (Doctor *et al*., 2014; Hale *et al*., 2014; Hecht *et al*., 2015; Pearse *et al*., 2014; Phillis *et al*., 2016). An *O. mykiss* population (Scott Creek, California; 37° 28′ N, 121° 56′ W), which was translocated in 1910 from below to above a waterfall, was shown to have undergone a 49% reduction in the frequency of the MAR migratory genotype (Pearse *et al*., 2014). However, lacustrine–adfluvial *O. mykiss* in artificial reservoirs, the dams of which were constructed in the latter half of the 19th century and prevent anadromy, share a high frequency of the MAR migratory genotype with anadromous stocks (Leitwein *et al*., 2017; Pearse & Campbell, 2018). Arostegui *et al*. (2019) found the same MAR genotype association in lacustrine–adfluvial *O. mykiss* in an Alaskan lake relative to river‐residents. Thus, in spite of the environmental differences, lacustrine–adfluvial and anadromous life histories appear to select for the same adaptive genomic variants on *Omy5*, providing further evidence of shared genetic control in both types of migration.

Lemopoulos *et al*. (2018) screened over 5500 SNPs for signatures of selection related to lacustrine–adfluvial migratory *v*. river‐residency in *S. trutta* in two catchments in Finland. Interestingly, four of the eight outlier SNPs mapped to genes previously shown to be involved in anadromy in salmonines with the three others being associated with genes involved in temperature changes and feeding.

### 3.2 Physiological condition

Numerous salmonid studies have linked many facets of an individual's physiological condition with the decision to migrate or remain resident, including aspects such as energetic state, metabolic rate and lipid storage (adiposity) levels (Ferguson *et al*., 2017). These physiological features of condition can be influenced by abiotic environmental factors (*e.g*., temperature, water flow), including those experienced during embryo development (Jonsson & Jonsson, 2014a), as well as by biotic factors such as food availability. There may also be individual heritable differences in the ability to acquire and utilise food resources (Van Leeuwen *et al*., 2015) as well as metabolic rate and efficiency (McMillan *et al*., 2012; Sloat & Reeves, 2014). Food availability, and thus energy limitation, has been shown to be closely associated with the migratory decision in salmonines, where the number of migrants, both lacustrine–adfluvial and anadromous, can be increased by reducing, directly or indirectly, the amount of food that individuals potentially receive (Archer *et al*., [Link]; Jones *et al*., 2015; Marco‐Rius *et al*., 2013; O'Neal & Stanford, 2011; Olsson *et al*., 2006; Wysujack *et al*., 2009). It is also important to acknowledge that food quality (*i.e*. energy value) may be as important as food quantity (Kendall *et al*., 2015). Given the relation with food availability, it might be expected that an increase in juvenile density would lead to a greater propensity for migration if competition for resources is present. Montorio *et al*. (2018) found that for *S. trutta*, first‐year density showed no correlation with migration although it correlated negatively with first‐winter survival and body size; the latter potentially resulting in delayed migration (see below). However, *S. salar* density was found to be positively correlated with *S. trutta* migration, indicating interspecific competition.

Temperature appears to be a key abiotic factor in the migratory decision (Brannon *et al*., 2004; Sloat & Reeves, 2014), with both absolute temperature and variation in temperature being important (Kendall *et al*., 2015). Temperature is clearly linked to food availability, feeding activity, metabolism and lipid storage and may also have a direct influence as a stressor on the migratory decision (Sogard *et al*., 2012). Peiman *et al*. (2017) examined the effects on *S. trutta* migration of experimental manipulations of temperature, food removal and cortisol administration, the latter mimicking a physiological challenge. They found that smaller individuals and individuals in poorer condition had a higher inclination to migrate and migrated earlier. Administration of cortisol, a key glucocorticoid, had the largest negative effect on growth and condition and resulted in earlier migration. However, Jain‐Schlaepfer *et al*. (2018) found no evidence indicating that cortisol is involved in the regulation of migration *v*. residency in *S. trutta*, but intermediate increases in baseline cortisol were correlated with increased survival during anadromous migration. Birnie‐Gauvin *et al*. (2019) also found baseline cortisol to be associated with the migration timing and success of anadromous *S. trutta* kelts. Birnie‐Gauvin *et al*. (2017) found antioxidant capacity to be associated with migratory status and migratory timing in *S. trutta*, several months ahead of actual migration. Migrants showed higher antioxidant capacity than river‐residents and within migrants, individuals with higher antioxidant capacity migrated sooner. This higher antioxidant capacity could enable migratory individuals to deal better with the energetic demands of migration (Birnie‐Gauvin *et al*., 2017).

## 4 INTEGRATING GENETIC AND ENVIRONMENTAL DETERMINANTS: THE THRESHOLD‐TRAIT MODEL

**Figure 2:**
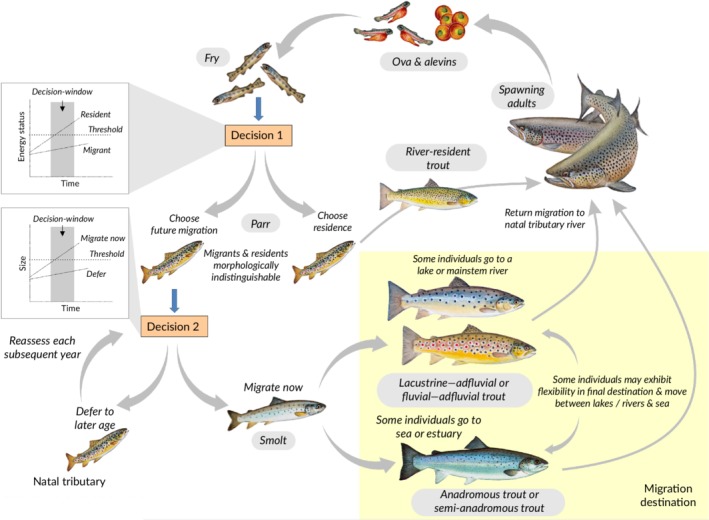
Potential life‐cycle diversity of Salmo trutta spawning in a tributary river with thresholds for migration–residency and age of migration. Note that the lake‐resident and allacustrine life cycles are not included

Facultative migration can be considered as a classic quantitative genetic trait; *i.e*., controlled by multiple genes and environmental factors. Unlike other quantitative traits that result in continuous phenotypes, the life‐history decision is dichotomous with the life history being one of alternative options controlled by a threshold; *i.e*., a typical threshold trait (Roff, 1996). This has been variously described in the literature as an environmental threshold model or a genetic threshold model (Cobben & van Noordwijk, 2017; Pulido, 2011), albeit both aspects are involved. Thus, the decision involves two components (Pulido, 2011): a liability trait or cue (a normally distributed trait describing some aspect of the individual's condition controlled by environmental signals and genes) and a genetically determined threshold for that condition, which determines the migration–residency decision. As outlined in §3.2, physiological condition is closely related to that decision. Thus, if an individual's energy status is sufficiently high to exceed its threshold value, the individual remains resident and becomes sexually mature. On the other hand, if it is nutritionally deficient and its physiological condition is lower than the threshold it prepares for migration, although it may not actually migrate for some time (Figure 2). Thus, the same genotype can result in different migratory traits as a result of environmental variability producing variation in the physiological condition cue; *i.e*., life‐history plasticity. Conversely, the same environmental conditions can result in different migratory traits due to genetic variability in the threshold and in the genetic component of the cue.

**Figure 3:**
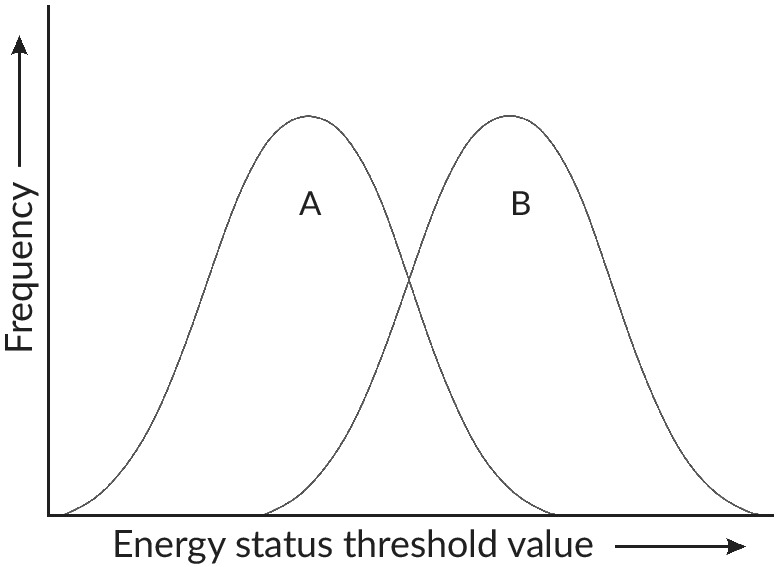
Theoretical distributions of physiological condition threshold values in males (A) and females (B), or two populations (A and B) of Salmo trutta with lower (A) and higher (B) propensities for anadromy. A single point along the *x*‐axis here corresponds to a single threshold value; *e.g*., the dashed threshold line at Decision 1 of Figure 2

It is assumed that individuals within a *S. trutta* population have different threshold values, which are likely to be continuous and follow a normal distribution, as is typical of other quantitative traits (Tomkins & Hazel, 2007), with mean threshold values differing among populations (Piché *et al*., 2008) (Figure 3). The distribution and mean threshold values are also expected to differ between sexes within a population, explaining their differential migration. Variation in threshold values means that the proportion of individuals expressing migration *v*. residency depends on both the distribution of variation in threshold values and the distribution of the physiological condition of individuals in the population. By extension, the threshold model can also be used to explain residency and obligate migration through differences in threshold values (Brönmark *et al*., 2014) with all or none of the individuals, respectively, achieving the threshold value for maturation as residents.

Threshold‐type models underpinned by state‐dependent life‐history theory have also been applied to understand variable life‐history trajectories in *S. salar*, in particular in relation to age of sexual maturity and age of smolt metamorphosis (Thorpe *et al*., 1998), although for males of this species it is a question of whether to mature before migration and not as alternatives as for *S. trutta*. In these *S. salar* models, maturation is assumed to be controlled by two developmental switches: one in early winter, a full year prior to spawning, the other in spring prior to spawning; smoltification is assumed to be controlled by an emigration switch occurring in the late summer, with the maturation switches dominating the emigration switch (*i.e*., if either is on, emigration does not occur the following spring and the fish remains on a freshwater maturation trajectory, a common outcome in male *S. salar*). A modified version of this model was applied by Sattertwaithe *et al*. (2009) to understand proximate and ultimate drivers of anadromous *v*. resident life histories in *O. mykiss* and while these models are conceptually related to the threshold models described here for *S. trutta*, there are some key differences. For example, while Thorpe *et al*. (1998) and Sattertwaithe *et al*. (2009) postulate the existence of separate, temporally distinct, maturation and smolt emigration switches, here a single early decision determining migration *v*. residency is assumed and a second later decision for the migrants determining age at smolting (Figure 2). Further extensions of the model, however, could include additional maturation‐timing decisions for both river residents and potamodromous/anadromous migrants, occurring in their respective habitats. While threshold models are compatible in general with much of what is known about the life‐history decision in facultatively anadromous salmonines (Ferguson *et al*., 2017; Kendall *et al*., 2015), further detailed mechanistic work is required to determine the number, nature and sequence of life‐history switches and other potential environmental and genetic mechanisms cannot be excluded at this time.

### 4.1 Does size matter?

In field experiments, various aspects of size such as length, growth rate, body mass and condition‐factor (length–mass ratio) are often used as surrogates for physiological condition, albeit the evidence for this association being inconclusive. Use of size as a surrogate potentially confuses two apparently separate thresholds. Thus, in *S. trutta* there appears to be an early threshold related to the decision to migrate and a second threshold linked to the actual timing (*i.e*., age) of migration, the latter remaining flexible for a longer period and potentially affected by environmental changes subsequent to the decision to migrate (Peiman *et al*., 2017). That is, individuals that have taken the decision to migrate may then have to pass a population‐specific size threshold before migration occurs, if not migration is deferred, resulting in migration occurring at different ages (Figure 2). Survival of migrants on entry to the new environment (*e.g*., lake or sea) may be positively size‐dependent in both *S. trutta* and *O. mykiss* (Klemetsen *et al*., 2003; Phillis *et al*., 2016), while larger fish that defer their migration may meanwhile fail to meet their higher energetic requirements in the river. Thus, larger fish at this second decision window should be selected to migrate now and smaller fish to defer, with the actual size threshold that evolves in a given population depending on the local selective pressures. In contrast, there is no reason for the initial migration *v*. residency decision, which occurs earlier in ontogeny, to involve a size threshold. Juveniles of a given genotype that encounter poor early feeding conditions are expected to be more likely to choose migration, but they could actually be smaller at this first decision window than other individuals that encountered better early feeding. Or there may be no obvious size difference between fish choosing migration *v*. residency at this point, despite differences in physiological condition. Confusingly, some studies appear to have been looking at the size threshold for timing of migration rather than the threshold for the migrate–mature decision (e.g. Phillis *et al*., 2016).

Not surprisingly then, size has been linked to the propensity for migration both positively and negatively and with evidence of population specific responses (Jonsson, 1985). Compared with residents, migratory *S. trutta* individuals have been found to be smaller (Morinville & Rasmussen, 2003) or larger (Acolas *et al*., 2012), have lower body mass (Winter *et al*., 2016), have lower condition‐factors (Boel *et al*., 2014; Wysujack *et al*., 2009) and have higher (Acolas *et al*., 2012) or lower (Morinville & Rasmussen, 2003) growth rates. Acolas *et al*. (2012) note that growth rate is a better predictor of migration than size, while Winter *et al*. (2016) claimed that body mass was better than length.

Conflicting results may also in part be due to failure in some studies to fully account for potentially confounding variables. For example, size at migration is unlikely to reflect size at decision time perhaps a year earlier (Acolas *et al*., 2012; Beakes *et al*., 2010; McKinney *et al*., 2015). In the meantime residents may have diverted energy from growth to sexual maturation. As survival in the early marine phase is size dependent (Klemetsen *et al*., 2003; Phillis *et al*., 2016), pre‐migrants may have accelerated growth during this period with the extent of growth being negatively correlated with size at last annulus (Thomson & Lyndon, 2018). That is, potentially the largest smolts may have been the smallest individuals at decision time. Emigration may occur over several successive years for the same cohort with some studies examining only 1 year, without any indication of whether non‐migrants could have migrated in subsequent years. Forseth *et al*. (1999) found that faster growing lacustrine–adfluvial *S. trutta* became migratory earlier, albeit at a smaller body size than slower growing individuals, which migrated 1 year later. It is sometimes overlooked that, in some populations, *S. trutta* smolt migration can occur in both the autumn and the spring and the size and mass of these two groups may differ, even though both groups appear equally successful in terms of returning anadromous *S. trutta* (Birnie‐Gauvin & Aarestrup, 2018; Winter *et al*., 2016). Failure to account for sex of juveniles and of their parents, can make it difficult to evaluate effects of size on migration in some studies. Males from resident *O. mykiss* mothers matured at smaller sizes than those from anadromous mothers (Berejikian *et al*., 2014). McMillan *et al*. (2012) found no difference in size between migrant and resident *O. mykiss* unless males and females were examined separately. Population‐specific patterns of genetic covariance among linked traits, such as size, growth rate, metabolic rate and age at migration or sexual maturity as residents, may also in part explain the contradiction among studies regarding the role that these traits play in the life‐history decision (Doctor *et al*., 2014; Dodson *et al*., 2013; Hecht *et al*., 2015).

## 5 MIGRATION DESTINATION

Once the decisions are taken regarding if and when to migrate the next decision is, the destination for adult feeding. As indicated by the results described below, while more attention has been given to the mechanisms of return spawning migration and natal homing (Bett & Hinch, 2016), various indirect lines of evidence suggest that out‐migration pathways in *S. trutta* and other salmonines are also genetically influenced. In passerine birds migration pathways to geographically distinct wintering areas are genetically encoded and specific genes associated with particular migratory phenotypes have been identified for some species (Lundberg *et al*., 2017). However, while salmonines show innate compass orientation in the marine phase (see below), it is not known if the resolution of the magnetic‐field map is sufficient to provide positional information over the more limited scale of a river catchment (Scanlan *et al*., 2018). In some situations, it is not a matter of moving downstream until the feeding destination is reached since, for some allacustrine populations where spawning occurs in a tributary of the outlet, getting to the lake requires downstream migration followed by upstream migration (Figure 1). It is difficult to envisage how this could be achieved without innate instructions. Allacustrine spawning salmonines must move upstream to reach the lake unlike downstream migrating lacustrine–adfluvial inlet spawners. Several common‐garden experimental studies on *S. trutta*, *O. mykiss* and cutthroat trout *Oncorhynchus clarkii* (Richardson 1837) have indicated that this, as with the downstream movement of inlet‐spawned offspring, is an inherited adaptive response to current direction (Jonsson *et al*., 1994; Kelso *et al*., 1981; Raleigh & Chapman, 1971).

In general, for a particular *S. trutta* population the feeding destination remains fixed from year to year although it can change over time as a result of natural selection due to alterations in costs *v*. benefits for migration to that particular habitat (see §7). The destination appears to be already decided when the migration begins. Anadromous *S. trutta* can move through both lake and downstream river habitats to reach the sea without any indication of stopping on route. Similarly, lacustrine–adfluvial *S. trutta* can migrate through other lakes to reach their destination lake (Huusko *et al*., 2018). Given that both mortality and energy expenditure of salmonid smolts are considerably increased in passage through lakes compared with rivers (Honkanen *et al*., 2018; Schwinn *et al*., 2018), it might be expected that *S. trutta* smolts would not continue through a lake unless programmed to do so. In coastal Californian rivers, summer sandbars at estuary mouths result in seasonally closing estuaries that form small productive freshwater lagoons. The migratory behaviour of *O. mykiss* in these rivers is governed by the availability of this seasonal habitat (Hayes *et al*., 2011) and appears to be adapted to these specific conditions. Migratory *O. mykiss* moved downstream in the spring and all displayed elevated Na^+^K^+^‐ATPase (NKA) activity levels. Larger fish (> 150 mm) moved downstream during February and March, leaving the river, whereas fish moving between April and June were typically smaller and stopped in the estuary, with NKA activity levels declining over the summer. The latter *O. mykiss* moved upstream in the autumn when estuarine conditions deteriorated but subsequently migrated to sea the following spring (Hayes *et al*., 2011).

Cucherousset *et al*. (2005) found that juvenile metabolic requirements and rate of growth, particularly in the second year, were important in determining if *S. trutta* remained resident or became fluvial–adfluvial or anadromous migrants. Individuals with a low metabolic rate remained in their natal tributaries as they could obtain sufficient food to meet their metabolic needs*. Salmo trutta* with higher metabolic needs migrated to a higher order tributary and if they were able to maintain their growth they remained there. If not, they extended their downstream migration to the main‐stem of the river or to the sea. Similarly, Boel *et al*. (2014) suggested that the time and distance that individual *S. trutta* migrate may be controlled by energy status. Thus, short‐distance lacustrine–adfluvial migrants were more lipid depleted than long‐distance, potentially anadromous, migrants that continued their migration through the lake. The fish with greater energy depletion apparently terminated their migration at the earliest increased feeding opportunity. These studies would suggest that environmental factors, such as food availability in relation to metabolic needs, play a part in determining migration destination. However, along with environmental factors, genes play a role in metabolic efficiency and energy status and thus may indirectly determine migration destination in these two catchments.

There is good evidence for genetic control of feeding location in the marine phase for anadromous salmonines with several species, including *S. trutta*, showing site fidelity for feeding location (Losee *et al*., 2018; Quéméré *et al*., 2016). In the Danish Limfjord (56° 55′ N, 09° 02′ E), anadromous *S. trutta* were found to exit by the original eastern outlet into the Kattegat rather than the western one into the North Sea even though the latter was formed in 1825, indicating likely adaptation to the eastern route (Kristensen *et al*., 2018). Hatchery‐reared anadromous *S. trutta* from different populations showed distinct migration pathways when released from the same site in the Baltic (Svärdson & Fagerström, 1982; Kallio‐Nyberg *et al*., 2002) and natural populations differed in their distribution at sea (Jonsson & Jonsson, 2014b), indicating at least a partial genetic basis for their migratory behaviour. Juvenile anadromous *O. mykiss*, without prior migratory experience, responded to magnetic fields at the latitudinal boundaries of their ocean range with oriented swimming that would lead them towards appropriate foraging grounds (Putman *et al*., 2014a) and recent work has identified candidate genes linked to magnetoreception (Arniella *et al*., 2018; Fitak *et al*., 2017). Two Chinook salmon *Oncorhynchus tshawytscha* (Walbaum 1792) populations and their hybrids, reared under identical conditions, differed in their oceanic distribution and hybrids displayed an intermediate distribution relative to the two pure populations (Quinn *et al*., 2011). Subsequently *O. tshawytscha* were shown to use an inherited magnetic map that facilitates navigation during their oceanic migration (Putman *et al*., 2014b)*. Salmo salar*, from a long‐standing non‐anadromous population, were shown to be able to orientate in novel magnetic fields (Scanlan *et al*., 2018). As this ability to extract location information from the Earth's magnetic field is present in at least three salmonines species, it seems to be an ancestral state in the sub‐family and thus is very likely to be present in *S. trutta*.

## 6 CAUSES AND CONSEQUENCES OF LIFE‐HISTORY DECISIONS

Fundamental to facultative migration is the decision on whether to migrate or to remain as a resident in the river and mature, which may take place a considerable time before external evidence of migration occurs (Hecht *et al*., 2015; McKinney *et al*., 2015). The switch between resident and migratory phenotypes is a complicated process involving sensing the cue, comparing it to an individual threshold, triggering a physiological or other response and development of that response (Buzatto *et al*., 2015). It is important to distinguish between the decision‐making process and the many subsequent responses activated by that decision. Failure to recognise that important distinction has led to misinterpretation of some studies. Studies at the smoltification stage (see §6.2) are the earliest at which it is possible to externally differentiate migrants from residents within a population and many comparative studies on smolts and non‐smolts have been undertaken for this practical reason. However, such studies primarily indicate the physiological and other changes necessary for migration or maturation and not with why the decision to migrate was taken in the first place. Similarly, it is important to distinguish between environmental factors involved in the migration decision from those that act as stimuli for the timing of the actual migration. Pirhonen *et al*. (1998) found that both anadromous and lacustrine–adfluvial *S. trutta* smolts migrated at the same time, suggesting that similar influences may be involved in their timing.

### 6.1 Gene regulation and epigenetics

The translation of the same genome into different phenotypes (phenotypic plasticity) requires differential gene expression. That is, the genotype does not unambiguously determine the phenotype but rather the range of phenotypes that can be produced under different environmental conditions. This is referred to as the reaction norm. Gene regulation involves various chemical messages that are responsible for switching individual genes on or off, thus facilitating or inhibiting the production of specific proteins, but without changing the underlying DNA sequence. Collectively these changes are referred to as epigenetic mechanisms, with the modified genome being referred to as the epigenome. However, the term epigenetic is often used inconsistently and is sometimes used synonymously with epigenetic inheritance, which is a separate process (Norouzitallab *et al*., 2019). DNA methylation, histone modifications and the activity of non‐coding–small RNAs, are the major mechanisms of epigenetic regulation in eukaryotes, although several other pathways are known (Villota‐Salazar *et al*., 2016). Across vertebrates, there are many examples where environmental effects experienced by parents, often very early in their own lives, that can be transmitted to their offspring, but the role of epigenetic inheritance, as defined here, in this remains unclear (Burton & Metcalfe, 2014). For this transgenerational epigenetic inheritance to happen, changes must occur in the gametes and avoid reprogramming or erasure in the embryo. Although few studies have so far been undertaken in salmonines, there are suggestions that some epigenetic changes in the parental genomes might be transmitted to their offspring. Many differentially methylated regions (DMR) have been shown to occur in the sperm of hatchery‐reared *v*. wild *O. mykiss*, demonstrating the potential for inheritance (Gavery *et al*., 2018). It should be noted that epigenetic inheritance, for example mediated *via* effects of temperature on gene regulation, could potentially influence the life‐history traits of offspring and perhaps grand‐offspring, irrespective of energy status (Jonsson & Jonsson, 2019).

A key but underappreciated aspect is that theory generally predicts (Bonduriansky & Day, 2009) that transgenerational non‐genetic effects, including the special case of epigenetic inheritance, should only be adaptive when there is some degree of predictability or autocorrelation between the parental and offspring (or grand‐offspring, *etc*.) environments. If the environment in generation *t* + 1 is uncorrelated or only weakly correlated with the environment in generation *t*, then trans‐generational inheritance of environmental effects will be of little adaptive value and could actually increase the likelihood of phenotype–environment mismatching (Burton & Metcalfe, 2014). Similar arguments apply to within‐generation phenotypic plasticity, which is only adaptive when the environment at the time of responding to some cue is correlated with the environment at the time of selection on induced phenotypes. A key question in the context of facultative migration in *S. trutta*, then, is whether such across‐generation environmental autocorrelation is present and strong enough to select for adaptive epigenetic inheritance to aspects of the decision process? Cyclical environmental phenomena such as climate oscillations could produce relevant environmental autocorrelation here, but so too could directional trends in environmental variables, *e.g*., associated with climate change.

The decision to migrate or remain resident will result in many epigenetic changes required for these distinct pathways. Several studies have demonstrated such epigenetic changes between resident and migrant *S. trutta* and other salmonines. Again, it should be emphasised that most differences found, especially at the smolt stage, are the consequence of the life‐history decision and not the cause of it. However, in a key common‐garden study, McKinney *et al*. (2015) examined changes in gene expression from hatching onwards involving the offspring of anadromous and resident *O. mykiss* from Sashin Creek reared under communal hatchery conditions for 1 year. They found differential gene expression in the brain between these lines for 1982 genes (7% of genes examined). Differences between anadromous and resident offspring were detected from hatching onwards with the greatest number of gene differences being found at 8 months of age, more than a year before obvious external appearance of smolt transformation. Patterns of gene expression during development differed between males and females, which may reflect the fact that males, in the resident population, mature earlier than females (McKinney *et al*., 2015). A caveat to the use of the offspring of allopatric anadromous and resident salmonines, in the McKinney *et al*. (2015) and other studies, is that aspects other than life‐history traits (including traits correlated with the anadromy decision, such as growth rate) may differ as a result of evolutionary divergence, although the recent common ancestry of the Sashin *O. mykiss* populations should minimise this.

Giger *et al*. (2006) found shared differences in the genes expressed among smolts and among resident *S. trutta*, from various European populations irrespective of their geographical and phylogenetic background, thus indicating common gene expression pathways related to smoltification and residency, as indeed is apparent across species. Giger *et al*. (2008) found that 21% of screened genes were differentially expressed in *S. trutta* smolts and non‐smolts, which would suggest that many genes are involved in smoltification, or are indirectly affected by the process, in keeping with the genome‐wide distribution of gene associations found in later studies. Many other studies have shown gene expression differences, especially in the gills, between smolts and resident salmonines (Houde *et al*., 2018; McKinney *et al*., 2015; Veale and Russello, 2017a). Baerwald *et al*. (2015) found 57 DMRs between smolt and resident *O. mykiss* juveniles derived from a cross reared under communal conditions. Genes that have been found to be differentially expressed relate, in most cases, to known physiological differences between smolts and residents; *i.e*., those associated with circadian rhythms, growth, homing, innate immunity, light sensitivity, metabolism, morphology, olfactory imprinting, osmoregulation and sexual maturation. Transaldolase 1 and endozopine are expressed at lower levels in both potamodromous and anadromous individuals compared with resident individuals and these differences can be detected some 3 months prior to migration (Amstutz *et al*., 2006; Giger *et al*., 2008); emphasising again the commonality of migration irrespective of destination.

### 6.2 Smoltification

Smoltification is a universal feature of all migratory salmonines and involves many changes including alterations to salmonid body shape and behaviour, silvering and changes to many enzymes and hormones, especially those produced by the thyroid (McCormick, 2013). Although often seen only as a preparation for anadromy, there is increasing evidence that many similar changes occur for potamodromous migrants. Thus, increase in NKA activity, widely used as an indicator of anadromous smoltification, occurs also in potamodromous migrants (Boel *et al*., 2014; Inatani *et al*., 2018), as do changes in behaviour, skin pigmentation and body morphology (Table 1). Whether this increase in NKA in potamodromous migrants has functional significance or simply reflects ancestral standing genetic variation is not known. NKA is composed of two structural subunits, α and β, together with a regulatory subunit, γ (Blanco & Mercer, 1998). In salmonines there are five isoforms of the NKA α subunit with α1a producing an NKA isozyme suited to fresh water and ion uptake and α1b suited to marine conditions and ion excretion (McCormick *et al*., 2009). Thus, in anadromous salmonines at parr‐to‐smolt transformation there is a switch in the α subunit composition, in addition to an overall increase in NKA activity, (McCormick *et al*., 2013). Downregulation of the α1a subunit and upregulation of the α1b occur while the fish are still in fresh water and this occurs prior to the increase in NKA activity in *S. trutta* (Seidelin *et al*., 2000), indicating that the migration destination is pre‐determined. Non‐anadromous *Oncorhynchus masou* (Brevoort 1856) were found to show an increase in NKA activity in smolt‐like individuals but, unlike anadromous individuals, this was not accompanied by an increase in the α1b isoform (Inatani *et al*., 2018).

A recent laboratory study of *S. trutta* showed that offspring of wild‐caught parents deriving from a naturally non‐anadromous population in Western Ireland displayed morphological signs of smoltification when exposed to reduced food supply as fry/parr, compared with fish from the same population experiencing optimal food rations (Archer *et al*., [Link]). However, putative smolts from this non‐anadromous population background exhibited reduced saltwater tolerance (as assessed by plasma chloride levels following 24 h of saltwater exposure) compared with smolts from a second population, which exhibits high rates of anadromy in the wild despite both sets of smolts having being raised under identical experimental conditions. These findings indicate that non‐anadromous wild populations of *S. trutta* may retain some genetic capacity for facultative anadromy, albeit with imperfect saltwater tolerance among resulting smolts, as has also been shown in *O. mykiss* (Phillis *et al*., 2016; Thrower *et al*., 2004).

Quantitative trait locus (QTL) studies show that variation in salinity tolerance among individuals of *S. salar*, *O. mykiss* and Arctic charr *Salvelinus alpinus* (L. 1758) has a genetic basis, with the same genes being involved in these species (Norman *et al*., 2012). The timing of smoltification is in response to environmental cues such as photoperiod (Strand *et al*., 2018), temperature (Haraldstad *et al*., 2017) and water flow (Jensen *et al*., 2012), with the brain being the main integrator of this information and thus, the main regulator of the process (McKinney *et al*., 2015). This occurs through interpretation of seasonal cycles, often *via* the effects of photoperiods on circadian rhythms (biological clocks) and through various hormones (Björnsson *et al*., 2011).

In *O. mykiss*, smoltification is regulated by a complex genetic network, including the large MAR on *Omy5* together with additional gene loci on chromosomes *Omy10*, *Omy12 and Omy14* (Hale *et al*., 2013; Pearse *et al*., 2014). Hecht *et al*. (2012, 2013) found the largest number of genes associated with parr–smolt transformation located on *Omy12*. Just as the MAR on *Omy5* may act as a master switch for the migration decision, so the genes on *Omy12* may be the major controllers of parr–smolt transformation in anadromous *O. mykiss*, again emphasising that these are two distinct processes.

### 6.3 Return migration

Out‐migration requires a subsequent in‐migration for spawning or, where adverse conditions occur at the migration destination, to a suitable refuge from harsh conditions. Non‐mature fluvial–adfluvial *S. trutta* in Spain have been shown to migrate upstream at times other than the main spawning run period, possibly for thermoregulation (García‐Vega *et al*., 2018). In Norway, where low winter sea temperatures occur, overwintering of immature anadromous *S. trutta* is often in fresh water (Klemetsen *et al*., 2003). This is possibly due to the marine hypo‐osmoregulatory capacity being compromised by low temperature, although, it may also reflect differing life‐history traits or individual genetic differences in osmoregulatory capacity, since not necessarily all *S. trutta* in a population exhibit the behaviour (Thomsen *et al*., 2007) or individual genetic differences in osmoregulatory capacity. Thus, there are population differences in the expression of key stress and osmoregulatory genes suggesting that some populations may be more adapted to remaining at sea overwinter than others (Larsen *et al*., 2008) and there may also be individual heritable differences within populations. Where the return is for overwintering without spawning, both natal and non‐natal rivers are used. Anadromous *S. trutta* in Norway have been recorded wintering up to four times in other rivers before returning to their natal one for reproduction (Jensen *et al*., 2015). Studies on *S. alpinus* indicate that they overwinter in the closest rivers with the least energetically demanding migratory route, thereby potentially minimizing the migration costs in nonbreeding years (Moore *et al*., 2017). Overwintering in non‐natal rivers means that individual movements of physically tagged *S. trutta* overestimate the extent of actual gene flow among populations in different catchments (Masson *et al*., 2018) and that samples of older post‐smolt individuals are inappropriate as baseline samples in genetic assignment studies (Moore *et al*., 2017).

Return for spawning is generally to the natal river with this homing being undertaken with considerable accuracy as shown by the typical population genetic structuring of both anadromous and potamodromous *S. trutta* (Ferguson, 1989; Prodöhl *et al*., 2017). Longer distance homing at sea likely involves geomagnetic fields but closer to the home catchment and within the catchment, olfactory cues derived from the chemical composition of the natal river or population‐specific pheromones are important (Bett & Hinch, 2016). Social interactions; *i.e*., migrating as a group, may also play a part in navigation (Berdahl *et al*., 2014).

The age at which maturation and spawning migrations occur, together with the time of year and ultimate location of spawning vary among individuals within populations and among populations of salmonines, including *S. trutta* (Klemetsen *et al*., 2003; Quinn *et al*., 2016). Variation in all of these aspects has been shown to have significant genetic components consistent with evidence of local adaptation in these life‐history traits. Thus, age of maturation in *S. trutta* and other salmonines is a quantitative trait (Palm & Ryman, 1999) with a moderate heritability (Dodson *et al*., 2013; Reed *et al*., 2018). Variation in the incidence of maturation of male *S. salar* as parr has also been shown to be substantially genetically controlled (Lepais *et al*., 2017). In *S. salar*, a single locus containing the *vgll3* gene, with sex‐dependent dominance, has been shown to explain 39% of the differences in sea age at maturity (Ayllon *et al*., 2015; Barson *et al*., 2015). This locus has also been linked with iteroparity, with the early maturing genotype being more likely to reproduce again (Aykanat *et al*., 2019). Interestingly, the *vgll3* locus has previously been found to be associated with the timing of puberty in humans, suggesting a conserved mechanism for timing of maturation in vertebrates (Kjærner‐Semb *et al*., 2018). It would therefore seem highly likely that the same gene region may control time of maturity in *S. trutta*. Indeed, the two non‐synonymous substitutions identified in *vgll3* in *S. salar* are also present in *S. trutta* (Ayllon *et al*., 2015).

Although return migration timing within the year can be closely associated with spawning time, this is not always the case. Return migration timing has been shown to have a heritable basis in many salmonid species (Cauwelier *et al*., 2017) with clock genes being a significant contributor to this (O'Malley *et al*., 2010). Environmental variables are also involved with temperature during embryogenesis, for example, having been shown to influence exact seasonal timing of adult return to fresh water in *S. salar* (Jonsson & Jonsson, 2018). The effects of winter temperature experienced at the embryo stage on adult return timing could be an effective mechanism to allow return to the natal river at the correct time for spawning (Jonsson & Jonsson, 2019). There is also evidence of heritable components in time of spawning with, for example, strong correlation among family members of anadromous *O. mykiss* in respect of the exact day of spawning (Abadía‐Cardoso *et al*., 2013).

Anadromous *O. mykiss* and *O. tshawytscha* show bimodal timing of return to fresh water with non‐mature fish often returning many months prior to mature fish, which return closer to actual spawning time. However, while the time of arrival at the spawning grounds differs, both run‐time types can spawn at the same time and place with interbreeding resulting in lack of significant differentiation at neutral gene markers (Prince *et al*., 2017). A single diallelic locus (GREB1L), an oestrogen target‐gene, was shown to be closely associated with this migration difference in these species (Prince *et al*., 2017; Thompson *et al*., 2019) suggesting a relatively simple genetic basis for run‐timing. However, in a denser gene mapping study, Micheletti *et al*. (2018a) found that GREB1L is part of a larger genomic region under selection consisting of four genes on chromosome *Omy28* with a major effect on maturation timing and spawning ground arrival timing. Narum *et al*. (2018) found consistent association of the two run phenotypes with this genomic region across three distinct phylogenetic lineages. The occurrence of these distinct types in many populations of *O. mykiss* and *O. tshawytscha* appears to be the result of pre–existing genetic variation at this gene locus, which has spread by migration and positive selection, thus questioning the previous paradigm that these traits had arisen by parallel evolution in each population (Prince *et al*., 2017).

The place of spawning is also genetically controlled. Veale and Russello (2017a,b) found alleles associated with river‐spawning and lake shore‐spawning in *O. nerka* across their pan‐Pacific distribution, involving genetic variation within, or linked to, the region surrounding the *lrrc9* gene. In Lake Garda, the distinctive deep‐water lake spawning behaviour of the native *S. trutta* results in reproductive isolation from introduced *S. trutta*, which are lacustrine–adfluvial spawners (Meraner & Gandolfi, 2018), emphasising that their spawning locality is genetically determined, with adaptations probably being required for spawning at such depth and pressure.

## 7 DIRECT AND EVOLUTIONARY EFFECTS OF ENVIRONMENTAL CHANGES ON MIGRATION

**Figure 4:**
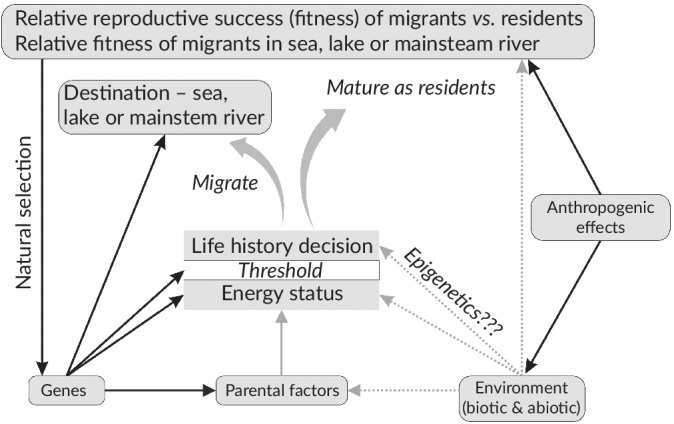
Summary of how genetic, environmental and parental factors could interact to determine the life history of Salmo trutta and how evolutionary changes to life history could result from environmental changes that alter the relative reproductive success of migration to a particular habitat *v*. river residency

All aspects associated with migration have been shown, in one or more salmonine species, to have an underlying genetic component and increasingly the specific genes involved or associated are being identified. That is, there is an overall migratory‐gene package, although it is also important to consider genetic covariation among traits since selection on one trait may indirectly result in selection responses in others (Liedvogel *et al*., 2011). Thus, all traits are potentially open to evolutionary changes under the action of natural selection with both natural differences in environmental conditions and anthropogenic induced alterations acting as agents of selection (Figure 4).

Unlike obligate anadromous salmonines, such as most populations of *S. salar* (Hutchings *et al*., 2019), facultative migration of *S. trutta* only occurs when the benefits exceed the costs, that is, where it leads to greater Darwinian fitness in terms of offspring production. Variability in the extent and destination of migration within populations and among geographically adjacent populations, would suggest that the relative benefits and costs are finely balanced and, therefore, evolutionary changes may occur rapidly as a result of relatively small alterations to the underlying factors. As environmental conditions change in the natal river, on the migration route and at the destination, so too will the benefits and costs of migration. When considering changes to facultative migration it is important to differentiate between direct (proximate) effects of environmental changes and indirect (ultimate) changes, operating through changes to fitness and thus natural selection and resulting in adaptive evolutionary changes. Environmental changes can influence the migration decision of an individual directly through changes to physiological condition. Alternatively, such changes can ultimately affect future generations through selection acting on the threshold or on the genes influencing physiological condition, with changes potentially occurring over a few generations with strong directional selection (Phillis *et al*., 2016). An increase in mortality of migrants can lead directly to reductions in the fraction of migratory fish through reductions in the number of subsequent spawners (proximate effect). It can also result in indirect changes in migration propensity, or in migration destination, across generations resulting from selection on underlying genetic mechanisms (ultimate effect). In practice, it is difficult to differentiate between proximate and ultimate effects in studies of natural populations as both processes act concurrently. However, while an individual *S. trutta* directly experiences the environmental conditions in its natal area, it has no way of assessing what the current conditions are on its migration route or at its destination habitat. Thus, its migration decisions are informed not by present conditions, but by the Darwinian fitness of its ancestors that drove either positive or negative natural selection on the migration threshold or genes determining destination.

While traits resulting in the highest overall fitness should in theory become fixed within a population, mechanisms collectively known as balancing selection may lead to the long‐term evolutionary maintenance of trait variation within populations. For example, fluctuating selection occurs when relative benefits and costs vary temporally because of diverse factors including different environmental conditions, population density and composition. Frequency‐dependent selection may also operate where increased frequency of one life history could allow selection to favour another until a balance is achieved (Hecht *et al*., 2015). Thus, as the migratory fraction increases, the remaining resident fish will have reduced competition for food, which may be advantageous even where these resources are less than would have become available through migration. Similarly, the rarer male type may have a competitive advantage in spawning. These aspects are explored in more detail in Table 2.

**Table 2:** Some non‐mutually exclusive hypotheses to explain why potamodromous and anadromous migrations of Salmo trutta are facultative rather than obligate

**Ecological conditions vary across time**
If the relative fitness of migratory and resident individuals varies through time, temporally fluctuating selection may favour the capacity of individuals to produce either type depending on physiological condition relative to a genetic threshold or bet‐hedging (where tactics develop randomly). *Examples: In some years, or for some cohorts, relative growth and survival benefits downstream, in a lake or at sea may outweigh those in the natal river, but in other years, the reverse may be true. Thus, neither tactic outcompetes the other in the long‐run*.
**Ecological conditions vary across space**
If the relative fitness of migratory and resident tactics varies across habitat types within a single freely interbreeding population, this may select for individuals that are capable of producing either tactic. *Examples: Fry that rear in more productive parts of the river, or that obtain better feeding territories, may be better off remaining resident and maturing early, whereas fry that rear in lower‐energy environments may gain more by becoming migratory. Smaller tributaries or spawning areas with smaller gravels may select for smaller resident females, whereas larger tributaries or areas with larger gravels may favour larger migratory females. A relatively small amount of gene flow among habitats/tributaries within rivers will still be enough to prevent genetic differentiation at neutral markers and possibly also at adaptive markers underpinning migratory decisions. But even in the absence of any spatial genetic differentiation within catchments, spatial variation in ecological conditions coupled with dispersal can select for conditional strategies (*Moran 1992 *) and thereby produce spatial variation in migratory tactics*.
**Frequency dependence favours a stable mix of tactics**
Smaller resident males may ‘sneak’ more fertilisations when rare, whereas larger migratory males may obtain more fertilisations on average when small resident males are most abundant. This mechanism can act to stabilise tactic frequencies at some intermediate value or, in theory, could lead to constant cycling of tactic frequencies. *Examples*: *Early maturing resident males have a spawning advantage relative to migratory males only when rare*.
**Sexually‐antagonistic selection maintains genetic variation in anadromy**
The evolutionary interests of males and females may be in conflict, such that genes that increase the propensity for migration are selected for in females but against in males. This then maintains genetic variation in the propensity for migration. *Examples: Females carrying genes for higher condition‐thresholds are more likely to be migratory, which increases their reproductive success, but their sons may then inherit these same genes and hence also become migratory, which may be less optimal for males than residency. Such ‘sexual conflict’ may mean that neither tactic has superior fitness overall (averaged across males and females), hence both co‐exist*.
**Heterozygote advantage favours the maintenance of genetic variation in anadromy**
For a given genetic locus affecting the propensity for migration, two or more alleles can be maintained in the population by balancing selection if heterozygotes have higher fitness than homozygotes. *Example*: *Heterozygous parents produce a mix of migratory and resident offspring, whereas homozygous parents produce more of one type than the other. If selection on average favours some intermediate threshold for migration, heterozygotes may have a long‐term fitness advantage over homozygotes. This mechanism could partially explain why genetic variation in migratory thresholds is maintained, but by itself does not explain why an intermediate degree of anadromy is favoured (although the other hypotheses might*).
**Optimal feeding destination may change over time**
If the genes determining the migration decision and the migration destination are linked they will co‐vary and not evolve independently. *Example: Feeding at sea may be best at one time but in a lake at another time, thus preventing genes responsible for the decision and destination being fixed. This is a special case of Hypothesis 1 above, with the added twist of genetic trade‐offs among traits (migration decision versus destination) due to pleiotropy (where the same genes affect multiple traits). Theoretical considerations, however, suggest that antagonistic pleiotropy may maintain genetic polymorphism only under a rather restrictive range of conditions (*Hedrick, 1999 *), so this mechanism may be less important relative to the others*.

If migration or river‐residency is advantageous in particular situations, it would be expected that compensatory adaptations would occur to increase benefits relative to costs (Hendry *et al*., 2004). Jonsson and Jonsson (2006b) found that anadromous *S. trutta* body size, age at sexual maturity, relative fecundity and the ratio of fecundity to egg mass increased with distance from the sea to the spawning grounds, consistent with the hypothesis that selection favours a larger body size when migratory costs are greater. Micheletti *et al*. (2018b) found evidence that the environment on the migration route of migratory *O. mykiss* can lead to substantial divergent selection, which varied on a regional basis. Migration distance to the sea and mean annual precipitation along the route were significantly associated with adaptive genetic variation. Additional variables such as minimum water temperature during migration and mean migration elevation were significant only in long‐distance migratory inland stocks. Adaptive variation associated with migratory landscape features was considerably greater than that associated with natal‐site landscape features. Distance from the feeding habitat to the spawning ground is an indicator of migration costs in terms of energy expenditure and mortality in migratory fishes. The time required for migration for a given distance is also likely to be an important factor in energy expenditure and is not simply related to distance but to barriers, presence of lakes, *etc*. (Table 3). However, altitude and distance of migration are negatively correlated with anadromy in *S. trutta* (Jonsson & Jonsson, 2006b; Ruokonen *et al*., 2018). The migration distance to reach the feeding destination coupled with the difficulties of the return migration (*i.e*., migration harshness) have been shown to have a strong effect on the bioenergetic costs involved with anadromous salmonines that migrated longer distances being more efficient in energy use than short‐distance migrants (Bernatchez & Dodson, 1987). Apgar *et al*. (2017) found that the anadromy associated MAR haplotype in *O. mykiss* declined with distance from the sea. In white‐spotted charr *Salvelinus leucomaenis* (Pallas 1814) and *O. masou*, where all females are anadromous but males show facultative anadromy, Sahashi and Morita (2013) used size as a proxy for the threshold value that determines migration. They found a decreased size at maturity with increasing distance from the sea; *i.e*., more males matured as residents. Size also covaried for the two species among 10 tributaries of a catchment covering a range of 100 km, consistent with either convergent evolution or convergent plastic responses.

**Table 3:** Anthropogenic factors potentially resulting in fitness changes and thus alterations to the cost–benefits of migration *v*. residency or migration destination in Salmo trutta and other salmonines

Factor	Impact on migrants	References
Partial barriers to downstream and upstream migration resulting from water offtake, hydroelectric generation, *etc*.	Increased energy expenditure. Increased risk of predation. Migration speed of smolts significantly slower. High downstream passage mortality of *S. trutta* kelts at hydropower stations. Upstream may be size selective and thus change size–age at maturity. Multiple partial barriers have an effect equivalent to an impassable barrier. Partial barriers resulting in a reduction of MAR alleles in *Oncorhynchus mykiss*. Removal of six partially impassable weirs in a Danish river resulted in nine‐fold increase in spawning *S. trutta* over 12 year period.	Apgar *et al*., 2017; Birnie‐Gauvin *et al*., 2018; Buddendorf *et al*., 2019; Haugen, 2008; Huusko *et al*., 2017; Jepsen *et al*., 1998; Ostergren & Rivinoja, 2008; Van Puijenbroek *et al*., 2018;
Complete barrier to upstream migration resulting from; *e.g*., construction of water storage reservoirs and hydropower stations without fish passes.	Anadromous populations extinct. Most of 72 anadromous *S. trutta* populations in Finland now lost. Change in destination; *e.g*., anadromous become lacustrine‐adfluvial migrants.	Holecek & Scarnecchia, 2013; Leitwein *et al*., 2017; Soininen *et al*., 2018
Regulation of river flows. Also, redirection of water to hydropower stations.	Un‐naturally high and low flows resulting in decrease in or elimination of migrants. Delays and increased energy expenditure. Changes in speed of migration. Fluvial–adfluvial became river‐resident due to reduced habitat quality.	Garcia‐Vega *et al*., 2017; Sandlund & Jonsson, 2016
Increased infestation by sea lice *Lepeophtheirus salmonis* associated with *Salmo salar* farming.	Reduced marine survival with 50%–100% mortality within 15 km of farms in Norway. Problems with osmoregulation. Earlier return to rivers with lower growth and fewer offspring thus reducing advantage of migration.	Gargan *et al*., 2016; Halttunen *et al*. 2017; Moore *et al*., 2018; Poole *et al*., 2006; Skaala *et al*., 2014; Taranger *et al*., 2015; Thorstad *et al*., 2015;
Increased predation by piscivorous birds and mammals in downstream sections of rivers, in lakes, and at sea.	Reduced survival. Increased energy expenditure in predator avoidance. Greater increase in predation at sea tips balance in favour of potamodromy. Predation through lakes and on sea entry main factor determining number of returning anadromous *S. trutta* in Denmark. High predation by great cormorants *Phalacrocorax carbo* key mortality factor in some rivers & lakes. Heavy pike *Esox lucius* predation at river‐to‐lake confluences.	Berejikian *et al*., 2016; Healy *et al*., 2017; Jepsen *et al*., 2018, 2019; ; Kennedy *et al*., 2018; Schwinn *et al*., 2018
Increased exploitation. Differential life history, size, and sex exploitation.	Reduced marine survival due to exploitation either directly or as a by‐product. Greater exploitation of (larger) migrants than (smaller) river‐residents resulting in selection for latter. Selection for earlier age of maturity, run timing and time of spawning.	Czorlich *et al*., 2018; Hollins *et al*., 2018; Kallio‐Nyberg *et al*., 2018; Koeck *et al*., 2018; Syrjanen *et al*., 2018; Thériault *et al*., 2008; Tillotson & Quinn, 2018
Climate change.	Changes in river flows and water temperature influencing feeding, migration timing, spawning and juvenile survival. Increased metabolic cost of upstream migration. Decreased marine productivity and increased freshwater productivity and growth rates tipping balance in favour of potamodromy–river‐residency. Possibly direct effect of temperature on life history.	Finstad & Hein, 2012; García‐Vega *et al*., 2018; Hermoso & Clavero, 2011; Jonsson & Jonsson, 2011, 2019; ; Lennox *et al*., 2018; Peiman *et al*., 2017; Piou & Prévost, 2012.
Interbreeding with stocked fertile hatchery reared / farm *S. trutta*.	Decreased genetic tendency for migration. Reduced marine cf. freshwater survival.	Ferguson, 2007; Ferguson *et al*., 2017; Thrower & Hard, 2009;

MAR: migration‐associated region.

For natural populations above upstream impassable barriers, there is clearly strong selection against migration since migrants are lost from the population, which results in genetic differences in respect of other life‐history aspects as well (Thrower *et al*., 2004; Thrower & Hard, 2009). As expected, in most studies of *S. trutta* and *O. mykiss* using genetic markers, there is no evidence of downstream gene flow from the above‐falls populations, although in a few cases there is evidence of limited active or passive movement (Ferguson *et al*., 2017). Anadromous traits may persist above barriers, despite strong natural selection against this trait because of phenotypic plasticity, or negative correlation with other traits, *e.g*., male maturation (Thrower *et al*., 2004; Phillis *et al*., 2016), or because some aspects of the migratory life history are selectively favoured despite the lack of access to the ocean. Once the barriers are removed or modified migration may be resumed as has been shown for several salmonid species with anadromous individuals arising from resident, fluvial–adfluvial or lacustrine–adfluvial ancestors (Archer *et al*., [Link], Quinn *et al*., 2017, Weigel *et al*., 2014); an important consideration in the restoration of extinct populations. Although the initial migrants can show poor smoltification and low marine survival (Archer *et al*., [Link]; Thrower *et al*., 2004), these aspects of the migratory syndrome are expected to improve over subsequent generations under the influence of natural selection.

In a common‐garden experiment involving offspring of an anadromous *O. mykiss* population and an above falls resident population derived from it in 1910 (Scott Creek), Phillis *et al*. (2016) found that the frequency of age 1+ year smolts in above‐barrier offspring was 54% compared with 73% in below‐barrier offspring and significantly more below‐barrier smolts were detected moving downstream compared with above‐barrier smolts. Seawater trials showed a 37% relative reduction in salinity tolerance in the above‐barrier offspring. Mature males accounted for 27.8% of all above‐barrier males but only 5.4% of below‐barrier males. These changes are consistent with natural selection for river residency in the *c*. 25 generations since establishment.

Sahashi and Morita (2018) examined how the migratory threshold size changed in response to opposing effects of natural and artificial selection in facultatively migratory male *O. masou*. In fish from above an impassable waterfall it was found that, in this high‐cost migration situation, the size threshold for migration had changed in the direction that promoted residency, relative to that in the below‐falls population. By contrast in the obligatorily resident *S. malma*, the size threshold did not differ in above and below‐waterfall populations indicating that environmental differences did not affect it. In a hatchery strain of *O. masou* that was subject to artificial selection for migration, the threshold altered in a way that favoured migration.

The numbers of returning anadromous *S. trutta* has declined over recent decades in many parts of north‐western Europe (Ferguson *et al*., 2019; Rasmussen *et al*., 2019). Similarly, lacustrine–adfluvial *S. trutta* have declined in some countries including Finland (Syrjanen *et al*., 2018) and Switzerland (Gafner & Meyer, 2018). The sustained nature of these declines means that genetic changes have probably occurred in response to changes in fitness and thus natural selection. Model projections by Satterthwaite *et al*. (2009) for anadromous *O. mykiss* suggest that if sea‐survival rates are reduced by some 50%, anadromy no longer occurs, although the extent of the reduction in survival required was population specific (Satterthwaite *et al*., 2010). The decrease in anadromous *S. trutta* numbers has involved multiple anthropocentric factors during both out and return migrations, as well as at the feeding destinations, which have resulted in both a reduction in survival of migrants or an increase in the costs of migration (Table 3). Thus, although, for example, many barriers do not stop downstream and upstream migration occurring there is cumulative increase in the likelihood of predation and a cumulative energy cost due to delays. The benefits of migration are reduced and the balance is potentially tipped in favour of river‐residency. This can also result in a change to the migration destination, for example, anadromous *S. trutta* become lacustrine–adfluvial migrants.

## 8 FURTHER GENETIC STUDIES

Studies on the genetic determinants of migration in *S. trutta* lag substantially behind those on other salmonines especially *O. mykiss*, although even for the latter species most studies have, until recently (Arostegui *et al*., 2019), only involved anadromy (Kendall *et al*., 2015). With current rapidly changing environmental conditions and diminishing numbers of individuals undertaking migratory life histories, studies of such determinants in *S. trutta* are urgently required. The development of genomic techniques now makes this feasible. Given the considerable similarities of facultative migration in the two species, *O. mykiss* genomic studies could act as a springboard enabling rapid progress to be made in respect of *S. trutta*.

Much more attention is required to be given to potamodromous migrations. Just as cross‐taxa comparisons can be informative (Dingle, 2006; Sahashi & Morita, 2013), so comparative studies of different migration patterns within *S. trutta* may be more informative than focusing on a single life history such as anadromy. It is emphasised that studies need to target early developmental stages, as this is when the migration– residency decision occurs and not focus exclusively on later stages such as smolts. This will require making use of experimental lines derived from river‐resident populations and populations with a high incidence of migration, together with innovative approaches, *e.g*., using associated genetic markers to predict the future life histories of individual fish at an early developmental stage. Studies need to be undertaken on all aspects of migration through to spawning of returning migrants. Sex needs to be taken into account in all studies, using genetic sex determination methods where necessary for juveniles.

Ecological, behavioural and physiological studies of the propensity for migration have often being carried out against variable genetic backgrounds where both intra‐population and inter‐population genetic variability was present, often with conflicting results. It is generally well recognised that examining genetic differences between populations or other groups requires studies to be carried out in communal environmental conditions (common‐garden experiments), with reciprocal hybrids to control for parental effects. However, it seems less widely appreciated that investigating the influence of varying environments requires either common gene‐pool experiments or reciprocal transfers of pure and hybrid stocks among the environments being tested.

Since variability in the migration– residency decision, migration destination and other aspects of migration involve substantial genetic components, empirical and modelling studies that ignore genetic aspects are of limited value. Fundamental to such studies are quantitative assessments of heritability and relative fitness of various components of migration and, in particular, residency *v*. potamodromous and anadromous decisions. Such estimates need to be undertaken in a range of *S. trutta* populations of different phylogeographic origins and biological characteristics (Ferguson, 2006) and across a range of environmental conditions within each population, where possible. To facilitate empirical studies, genetic variant makers for the migration–residency decision and other components of the migratory syndrome need to be determined. A good starting point would be the identification of *S. trutta* genes homologous to the *Omy*5 migration‐associated supergene region. Although it is not known if the same double inversion region is present in *S. trutta* (Pearse *et al*., 2018), it is likely that the same genes occur. The imminent availability of the *S. trutta* genomic sequence should make identification of these genes and associated SNPs, relatively straightforward. In addition, other salmonid chromosome regions–genes that have been shown above to be associated with migration should be investigated in *S. trutta*.

Validation of the threshold trait model for *S. trutta* is required together with elucidation of the structural and regulatory gene mechanisms underlying variation in the cue and the threshold. Also, are the decision and timing thresholds linked at the genetic level? An open question is whether the environment can influence migration other than through the threshold pathway (*i.e*., by its effect on physiological condition), or if it can influence gene regulation directly and perhaps be epigenetically inherited. A further unanswered question concerns the extent to which individual physiological condition and the migration–residency decision reflect adaptive responses of individuals to predictive cues, *v*. non‐adaptive, unavoidable constraints (Ferguson *et al*., 2017). Knowledge of the relative importance of such changes in producing alterations in migration is particularly important for restoration attempts. Thus, while proximate changes could be reversed very quickly by removing the adverse factor(s), ultimate changes are likely to take many generations. The role of genes in determining physiological condition, through their effect on metabolic rate and efficiency together with energy storage, should be investigated. That is, what determines the distribution of the cue? The physiological mechanisms by which the cue is perceived and compared with the threshold and that result in downstream regulatory gene changes should be established.

## AUTHOR CONTRIBUTIONS

This review derives from A.F.’s and T.F.C.’s interest and collaboration, over almost 50 years, in the genetics of life‐history diversity in *S. trutta* and *S. salar*, topics now being actively researched by T.E.R., P.McG. and P.A.P. A.F. prepared the first draft of the review, which was added to and revised by all authors through several further drafts. All authors approved the final manuscript.
